# Detection and Genomic Characterization of an Avian Influenza Virus Subtype H5N1 (Clade 2.3.4.4b) Strain Isolated from a Pelican in Peru

**DOI:** 10.1128/mra.00199-23

**Published:** 2023-05-01

**Authors:** Manolo Fernández-Díaz, Doris Villanueva-Pérez, Luis Tataje-Lavanda, Angela Montalvan-Avalos, Gisela Isasi-Rivas, Milagros Lulo-Vargas, Manolo Fernández-Sánchez

**Affiliations:** a Laboratorios de Investigación y Desarrollo, Farmacológicos Veterinarios S.A.C. (FARVET SAC), Chincha Alta, Ica, Peru; b Escuela Profesional de Medicina Humana, Universidad Privada San Juan Bautista, Lima, Peru; Queens College Department of Biology

## Abstract

Surveillance helps us identify and monitor strains with zoonotic potential. A tracheal swab from a pelican on a Peruvian beach was H5N1 positive (clade 2.3.4.4b) using Oxford Nanopore’s MinION platform. The near-complete genome sequence of strain VFAR-140 will aid us in understanding avian influenza epidemiology and spread.

## ANNOUNCEMENT

Highly pathogenic avian influenza (HPAI) caused by the H5N1 virus, a member of the family *Orthomyxoviridae* and genus *Influenza*, is an epidemic disease that causes significant economic losses (1). Seabird colonies, with their high population density, are particularly vulnerable to HPAI (2).

Tracheal swabs were collected from a dead pelican displaying respiratory symptoms consistent with avian influenza virus (AIV) in December 2022 in Tambo de Mora District (Chincha Province, Ica, Peru). The bird exhibited gasping, sneezing, and neurological symptoms such as opisthotonus. The swabs were analyzed at FARVET’s biosecurity level III (BSL-3) laboratory. A reverse transcription-quantitative PCR (qRT-PCR) assay targeting the M gene (3) was used to confirm the presence of AIV, while also screening for other respiratory viruses (4–6).

To isolate AIV H5N1 (VFAR-140), one positive tracheal swab sample was centrifuged (4,500 rpm); the supernatant was filtered (0.22 μm) and inoculated into 10-day-old specific pathogen-free (SPF) embryonated eggs. The allantoic fluid (AF) was collected, and the M (3), HA (7), and NA (8) genes were amplified for typing. A hemagglutination assay (9) was performed on the AF, and a titer of 1:512 was obtained. VFAR-140 was concentrated and purified from 200 mL of infected AF using ultracentrifugation (18,000 rpm for 16 h at 4°C), followed by 25% sucrose gradient ultracentrifugation (27,000 rpm for 6 h). VFAR-140 was then resuspended in 200 μL of 1× Dulbecco’s phosphate buffered saline (DPBS), and the RNA was extracted using the RNeasy Plus microkit (Qiagen). A cDNA library was generated using the direct cDNA sequencing kit (SQK-DCS109; Oxford Nanopore Technologies) and sequenced on the MinION Mk1b instrument (Oxford Nanopore Technologies) using the FLO-MIN106 flow cell (Oxford Nanopore Technologies).

**FIG 1:**
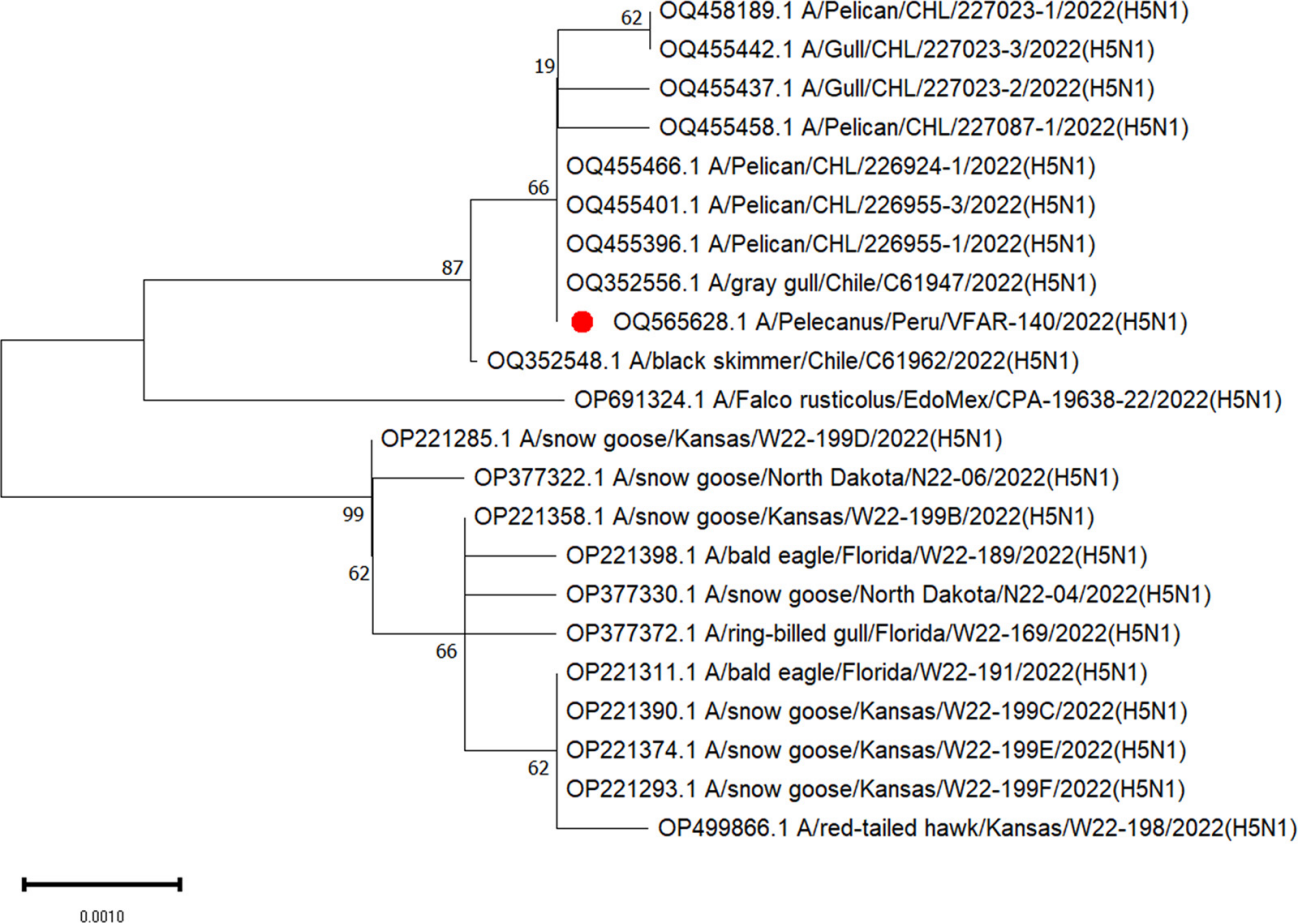
Phylogenetic tree based on coding-complete sequences (CDS) of the HA gene. The tree was obtained using the neighbor-joining method (TN93+G), with 1,000 bootstrap replicates (complete deletion). The analysis included 22 nucleotide sequences. Isolate VFAR-140 is marked with a red dot.

Default parameters were used for all software unless otherwise specified. Base calling was performed using Guppy v.6.3.7 (HAC model) (10). Fastq files were taxonomically assigned using the Fastq WIMP pipeline (11) with Kraken2 using the k2_standard_20210517 database (Galaxy v.2.0.8_beta+galaxy0) (12, 13) and visualized using Krona (Galaxy v.2.6.1.1) (12, 14). Adapters were trimmed using Porechop (Galaxy v.0.2.4+galaxy0) (12, 15). *De novo* assembly of all reads was performed using Raven (Galaxy v.1.8.0+galaxy0) (12, 16). A BLAST (17) analysis was used to identify four segments of AIV (segments 1, 2, 4, 5), and we selected a genome with complete coding sequences (A/gray gull/Chile/C61947/2022[H5N1]) as the reference genome. We subsequently mapped all reads against the reference sequence using BWA-MEM (Galaxy v.0.7.17.2) (12, 18) to obtain the final VFAR-140 genome sequences. The depth and coverage were determined using SAMtools (Galaxy v.1.15.1+galaxy0) (12, 19) and visualized using weeSAM v.1.6 (20). The identified positions were confirmed using BLASTn (17) analysis (Table 1). We obtained a total of 10,136 reads (*N*_50_, 3,353 bp; >Q5, 6,586 reads) from the isolate and successfully recovered eight segments of the VFAR-140 genome. Phylogenetic analysis of HA gene segment 4 was performed using MEGA v.11 (21) (Fig. 1).

**TABLE 1:** BLAST comparison results of nucleotide sequences of all segments of isolate VFAR-140 with those of closely related strains

Segment (gene[s])	Length (bp)	%GC	Depth of coverage (×)	Most closely related strain	Identity (%)	Reference sequence GenBank accession no.
1 (PB2)	2,338	45	71.96	A/pelican/CHL/226958-1/2022(H5N1)	99.23	OQ455420.1
2 (PB1; PB1-F2)	2,341	43	108.75	A/gray gull/Chile/C61947/2022(H5N1)	99.62	OQ352554.1
3 (PA; PA-X)	2,231	44	357.49	A/gray gull/Chile/C61947/2022(H5N1)	99.86	OQ352555.1
4 (HA)	1,754	41	63.23	A/gray gull/Chile/C61947/2022(H5N1)	99.66	OQ352556.1
5 (NP)	1,552	47	85.25	A/pelican/CHL/227023-1/2022(H5N1)	98.65	OQ455407.1
6 (NA^*a*^)	1,411	44	136.43	A/gull/CHL/227023-3/2022(H5N1)	99.72	OQ455402.1
7 (M2 and M1)	1,006	49	50.28	A/pelican/CHL/226955-1/2022(H5N1)	99.70	OQ455399.1
8 (NEP^*a*^; NS1)	814	46	62.51	A/Peruvian pelican/Chile/C61740/2022(H5N1)	99.75	OQ352544.1

a Partial coding sequence; all others listed are complete coding sequences.

The viral isolate VFAR-140 belongs to clade 2.3.4.4b H5N1 AIV, with the HPAI pathotype confirmed by the PLREKRRKRGLF cleavage site in HA (22). Molecular markers associated with increased polymerase activity in mice (23) were found in PB2 (L89V, G309D, T339K), and those associated with increased virulence in birds and mammals were found in PA (A515T) (24) and NS1 (P42S, V149A) (25, 26). However, no markers associated with mammalian adaptation were detected.

### Data availability.

The eight obtained segments were deposited in GenBank (accession numbers OQ565625–OQ565632). The raw sequence reads were deposited under SRA accession number SRR23852495. The sequences were also deposited in EpiFlu at GISAID (EPI_ISL_17099964: EPI2441726 to EPI2441733).
